# Benfotiamine improves dystrophic pathology and exercise capacity in *mdx* mice by reducing inflammation and fibrosis

**DOI:** 10.1093/hmg/ddae066

**Published:** 2024-05-06

**Authors:** Chantal A Coles, Keryn G Woodman, Elizabeth M Gibbs, Rachelle H Crosbie, Jason D White, Shireen R Lamandé

**Affiliations:** Murdoch Childrens Research Institute, The Royal Children’s Hospital, 50 Flemington Road, Parkville, Victoria 3052, Australia; Faculty of Veterinary and Agricultural Sciences, The University of Melbourne, Flemington Road, Parkville, Victoria 3052, Australia; Murdoch Childrens Research Institute, The Royal Children’s Hospital, 50 Flemington Road, Parkville, Victoria 3052, Australia; Faculty of Veterinary and Agricultural Sciences, The University of Melbourne, Flemington Road, Parkville, Victoria 3052, Australia; Department of Genetics, Yale Medical School, Yale University, 333 Cedar Street, New Haven, Connecticut 06520, USA; Department of Integrative Biology and Physiology, University of California, 612 Charles E Young Dr S, Los Angeles 90095, California, USA; Center for Duchenne Muscular Dystrophy, University of California, 615 Charles E Young Dr S, Los Angeles 90095, California, USA; Department of Integrative Biology and Physiology, University of California, 612 Charles E Young Dr S, Los Angeles 90095, California, USA; Center for Duchenne Muscular Dystrophy, University of California, 615 Charles E Young Dr S, Los Angeles 90095, California, USA; Department of Neurology, David Geffen School of Medicine, University of California, 610 Charles E Young Dr S, Los Angeles, California 90095, USA; Murdoch Childrens Research Institute, The Royal Children’s Hospital, 50 Flemington Road, Parkville, Victoria 3052, Australia; Faculty of Veterinary and Agricultural Sciences, The University of Melbourne, Flemington Road, Parkville, Victoria 3052, Australia; Charles Sturt University, Office of the Deputy Vice Chancellor Research, Boorooma Street, Wagga Wagga, NSW 2678, Australia; Murdoch Childrens Research Institute, The Royal Children’s Hospital, 50 Flemington Road, Parkville, Victoria 3052, Australia; Department of Paediatrics, University of Melbourne, 50 Flemington Road, Parkville, Victoria 3052, Australia

**Keywords:** Duchenne, muscular dystrophy, inflammation, benfotiamine

## Abstract

Duchenne Muscular Dystrophy (DMD) is a progressive and fatal neuromuscular disease. Cycles of myofibre degeneration and regeneration are hallmarks of the disease where immune cells infiltrate to repair damaged skeletal muscle. Benfotiamine is a lipid soluble precursor to thiamine, shown clinically to reduce inflammation in diabetic related complications. We assessed whether benfotiamine administration could reduce inflammation related dystrophic pathology. Benfotiamine (10 mg/kg/day) was fed to male *mdx* mice (n = 7) for 15 weeks from 4 weeks of age. Treated mice had an increased growth weight (5–7 weeks) and myofibre size at treatment completion. Markers of dystrophic pathology (area of damaged necrotic tissue, central nuclei) were reduced in benfotiamine *mdx* quadriceps. Grip strength was increased and improved exercise capacity was found in *mdx* treated with benfotiamine for 12 weeks, before being placed into individual cages and allowed access to an exercise wheel for 3 weeks*.* Global gene expression profiling (RNAseq) in the gastrocnemius revealed benfotiamine regulated signalling pathways relevant to dystrophic pathology (*Inflammatory Response, Myogenesis)* and fibrotic gene markers (*Col1a1, Col1a2, Col4a5, Col5a2, Col6a2, Col6a2, Col6a3, Lum*) towards wildtype levels. In addition, we observed a reduction in gene expression of inflammatory gene markers in the *quadriceps* (*Emr1, Cd163, Cd4, Cd8, Ifng*). Overall, these data suggest that benfotiamine reduces dystrophic pathology by acting on inflammatory and fibrotic gene markers and signalling pathways. Given benfotiamine’s excellent safety profile and current clinical use, it could be used in combination with glucocorticoids to treat DMD patients.

## Introduction

The muscular dystrophies are a group of genetic neuromuscular disorders that result in the progressive deterioration of skeletal muscle. Of these muscular dystrophies, Duchenne muscular dystrophy (DMD) is the most common affecting 1 in 5000 male births [1]. DMD results from mutations in the dystrophin gene leading to absence or severe reduction in dystrophin at the muscle plasma membrane [2]. Dystrophin is a critical component of a large complex known as the dystrophin- glycoprotein complex (DGC), present on the plasma membrane of the myofibre [3, 4]. Dystrophin stabilises cells by linking actin filaments, intermediate filaments and microtubules to transmembrane complexes, which interact with ligands in the extracellular matrix [5]. Loss of dystrophin, in most cases loss or reduction of the DGC leads to membrane instability, increased susceptibility to mechanical stress and finally, degeneration of myofibres [6]. Normal skeletal muscle possesses an innate ability to regenerate in response to injury. This is orchestrated by immune cells, satellite cells, fibroblasts and interaction with extracellular matrix. In dystrophic muscle, inflammatory cells, satellite cells & fibroblasts are continually activated as a result of chronic injury [7]. This pathological environment results in failed regeneration [8]. Consequently, myofibres are substituted by adipose/fibrotic tissue compromising muscle function.

Currently, glucocorticoid steroids (prednisone/prednisolone/deflazacourt) are the standard of care for DMD, increasing muscle function and prolonging ambulation in DMD boys. Ideally, the best treatment for DMD patients would be one that corrects the primary genetic defect. Recently, there have been pivotal advances in the gene therapy field with clinical trials of microdystrophin and exon skipping showing great promise [9–11]. However, these exon skipping trials in particular are only amenable to a particular subset of patient mutations and therefore there is a need for more treatment options. Given the contribution that secondary processes of inflammation, oxidative stress and fibrosis have in promoting DMD pathology, compounds that target these could be transitioned into the clinic.

Benfotiamine is a lipid soluble thiamine (vitamin B1) analogue with enhanced absorption and bioavailabilty compared to water-soluble thiamine [12, 13]. A single dose of benfotiamine can increase the plasma concentration of thiamine 5-fold compared to an equivalent dose of water soluble-thiamine [13]. Benfotiamine increases levels of thiamine derivatives in the blood and liver, but not in the brain [14–17]. Thiamine is not synthesised by humans and must be obtained by fortified foods or plant material in the diet. Benfotiamine functions during energy metabolism to facilitate thiamine diphosphate, a co-factor for transketolase, to accelerate precursors towards the pentose phosphate pathway [18]. Enhancing the action of transketolase becomes an important step in reduction and inhibition of advanced glycation end products (AGEs), which cause oxidative damage triggering an immune response [18]. In muscle tissue of the *mdx* mouse, high levels of the receptor for advanced glycation end products (RAGE) are present in areas of mononuclear infiltration, co-localising with macrophage markers [19]. Deletion of the RAGE receptor in *mdx* reduced muscle inflammation, caused macrophages to be less responsive to pro-inflammatory stimuli and improved muscle regeneration suggesting targeting RAGEs in dystrophic muscle has anti-inflammatory effects [19]. In addition, benfotiamine prevented LPS-activated release of arachidonic acid metabolites which are inflammatory mediators in macrophages [20].

Benfotiamine can also activate non-AGE dependent pathways to reduce oxidative stress [21] and activate Akt-dependant pro-survival signalling in heart, endothelial cells and skeletal muscle in diabetic mice [22–25]. Previous research has focused on benfotiamine as a therapeutic for a variety of diabetic related complications including cardiomyopathy [22, 23], retinopathy [26], limb ischaemia [24] and nephropathy [26, 27]. It has an excellent safety profile in humans and has been used in many clinical trials without adverse side effects [28–31]. Similarly, thiamine has been used in clinical trials for conditions such as gestational diabetic mellitus [32], cardiac arrest [33], COVID19 [34], sepsis and septic shock [33–35]. Considering benfotiamine’s ability to reduce oxidative stress and its anti-inflammatory role, particularly in macrophages which play a prominent role in dystrophic pathology, we tested its effect in the *mdx* mouse a pre-clinical model of Duchenne muscular dystrophy. In this study we treated dystrophic mice (*mdx*) with benfotiamine for 15 weeks. When compared to control *mdx* benfotiamine reduced multiple measures of dystrophic pathology, improved grip strength and voluntary exercise parameters by targeting gene markers of inflammation and fibrosis.

## Results

### Benfotiamine increases growth and promotes myofibre hypertrophy in mdx mice

**Figure 1:**
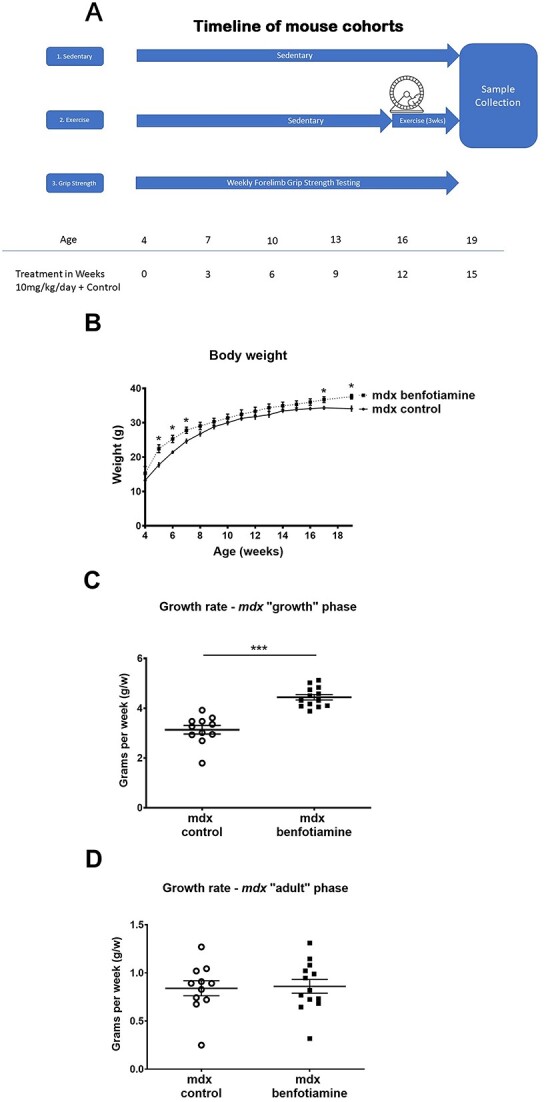
Timeline for mouse cohorts and growth data showing Benfotiamine increases *mdx* body weight and growth rate. (A) Schematic of timeline for three mouse cohorts used in study (B) sedentary from two independent experiments were weighed weekly for 15 weeks of benfotiamine treatment. Benfotiamine *mdx* mice weighed more than the *mdx* control from 5–7 weeks of age, then from 17–19 weeks of age. (C) The growth rate (grams per week) during the early *mdx* “growth” phase (4–8 weeks) was greater in benfotiamine *mdx.* (D) No difference in growth rate was found during the “adult” growth phase (9–15 weeks). The graphs show mean ± SEM. ^*^indicates *P* < 0.05 (benfotiamine n = 13 compared to *mdx* control n = 11: Mice are from cohort 1 and cohort 3 pooled).

We first interrogated the effect of benfotiamine on growth of *mdx* mice. Body weights of benfotiamine treated *mdx* and control *mdx* were compared over the treatment period starting at 4 weeks of age until completion at 19 weeks of age (Fig. 1B). Benfotiamine treated *mdx* mice were heavier than control *mdx* mice in the initial three weeks of treatment (ages 5 to 7 weeks) and at weeks 17 and 19 at the completion of the trial. To further investigate the change in weight we analysed the growth rate over the “growth” phase of mouse growth (4–8 weeks) and the “adult” phase (9–19 weeks). Benfotiamine treated *mdx* mice had a significantly increased growth rate during the “growth” phase (Fig. 1C) but not during the “adult phase” (Fig. 1D).

**Figure 2:**
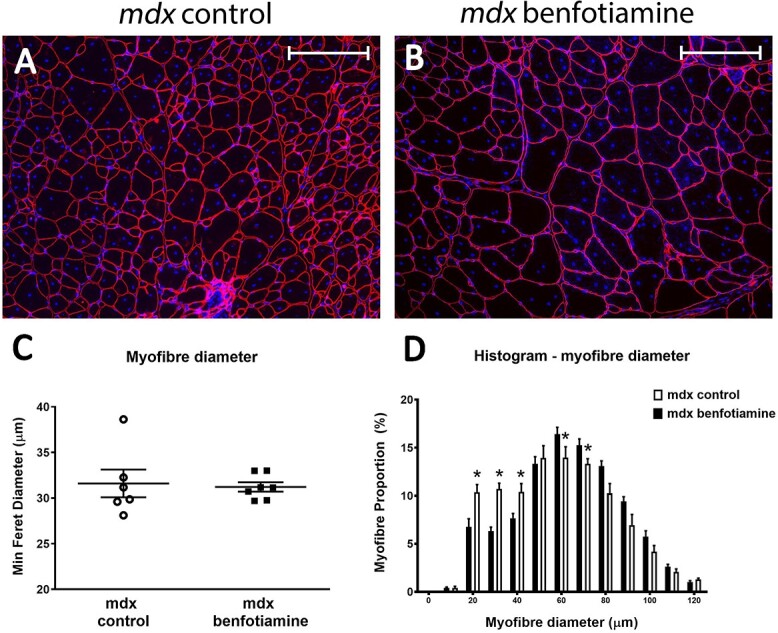
Benfotiamine increases myofibre size in *quadriceps* muscle. The quadriceps muscle from *mdx* control (A) and benfotiamine treated *mdx* (B) mice were stained with DAPI (blue) and laminin α2 antibody (red) to measure myofibre size (Feret’s minimum diameter). (C) Mean myofibre size in quadricep muscles was similar in untreated and benfotiamine treated *mdx.* Although no difference in mean myofiber size for minimum feret’s diameter, the coefficient of variation (% VC) was much higher in *mdx* (% VC 11.76%) compared with benfotiamine (% VC 4.35%) treated mice*.* (D) Frequency histogram showing there was a greater proportion of larger myofibres (70–90 μm diameter) and a reduced proportion of smaller myofibres (20–40 μm diameter) in benfotiamine *mdx* (D). ^*^P < 0.05, ^*^^*^P < 0.001, n = 6. Scale bar, 200 μm. *mdx* control n = 6 *mdx* benfotiamine n = 7.

To investigate benfotiamine’s effect on skeletal muscle, we first measured myofibre diameters. The mean myofibre diameter in benfotiamine treated *mdx* was not different compared with *mdx* control mice at 19 weeks of age (Fig. 2A–C). When we generated a frequency histogram for myofibres size, we found there was a decrease in the proportion of smaller myofibres (10–30 μm) in the quadriceps of benfotiamine treated *mdx* mice (Fig. 2D) (*P* < 0.05). Following muscle damage in dystrophic mice, regeneration occurs producing new, smaller myofibres. The benfotiamine mice did not have as many of these smaller myofibres and conversely, there was a greater proportion of large myofibres (70–80 μm) in the *mdx* benfotiamine treated quadriceps (Fig. 2D) (*P* < 0.05)*.* This result may be attributed to reduced damage in *mdx* benfotiamine treated.

### Benfotiamine reduces markers of dystrophic pathology

**Figure 3:**
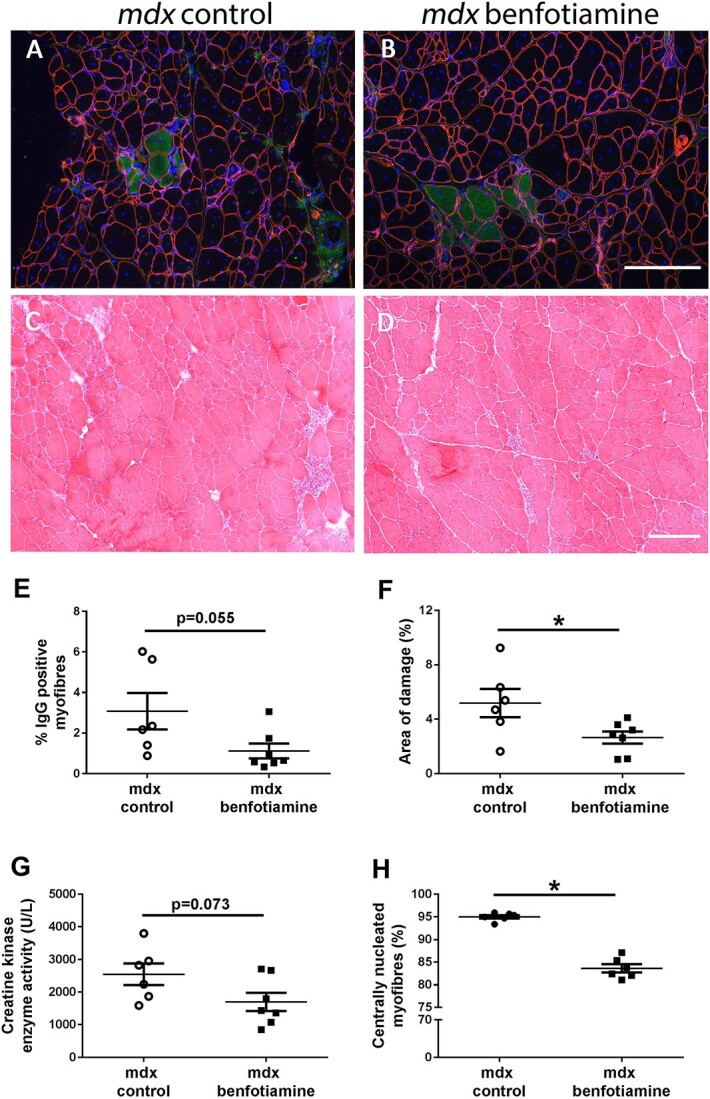
Benfotiamine reduces muscle damage and improves sarcolemma stability in *mdx* mice. Basal lamina (anti-laminin-α2 in red) and IgG (Alexa Fluor 488 in green) staining of transverse muscle sections were used to observe muscle integrity and damage respectively in *mdx* (A) and benfotiamine *mdx* (B) quadriceps. Nuclei are stained with DAPI (blue). Muscle architecture was visualised with hematoxylin and eosin in quadriceps of *mdx* (C) and benfotiamine *mdx* (D). (E) The percentage of damaged myofibres permeable to IgG in the quadriceps was not significantly reduced in benfotiamine *mdx* (*P* = 0.055). (F) Benfotiamine reduced the area of damage (including areas of necrosis and inflammatory cell infiltration compared to *mdx* control (*P* < 0.05). Muscle-specific creatine kinase in the serum, a marker of sarcolemmal damage, was not changed with benfotiamine treatment (G) (*P* = 0.073). (H) Benfotiamine reduced the proportion of fibres in *mdx* quadriceps muscle with central nucleation (*P* < 0.05). ^*^P < 0.05, *mdx* control n = 6 *mdx* benfotiamine n = 7. Scale bar, 200 μM.

Dystrophic pathology in DMD patients and *mdx* mice is characterized by elevated serum creatine kinase, myofibre degeneration, immune cell infiltration, fragmented muscle fibres and replacement of muscle with connective tissue. Transverse quadriceps sections were stained with anti-IgG to denote myofibres with compromised sarcolemmal integrity (Fig. 3A and B) and haematoxylin and eosin staining was used to visualize areas of necrosis (Fig. 3C and D). The percentage of anti-IgG positive myofibres was reduced by 55% in the quadriceps of benfotiamine treated *mdx* mice when compared to the *mdx* control mice ((*P* = 0.055) Fig. 3E). Serum creatine kinase (CK) activity, a measure of damaged muscle fibres was reduced by 33% in benfotiamine treated *mdx* mice but this was not significant (*P* = 0.073) (Fig. 3G). However, skeletal muscle necrosis (areas with infiltrating inflammatory cells and degenerating myofibres) was significantly reduced in benfotiamine treated *mdx* mice, when compared to the *mdx* control mice ((*P* < 0.05) Fig. 3F).

We also assessed the number of centrally located nuclei, a hallmark of muscle fibres that have undergone degeneration/regeneration cycles. Benfotiamine reduced the proportion of myofibres with centrally located nuclei in *mdx* quadriceps ((*P* < 0.05), Fig. 3H). Overall, assessment of these pathological markers demonstrates that benfotiamine reduced markers of muscle damage.

### Muscle strength and exercise is improved with benfotiamine treatment

**Figure 4:**
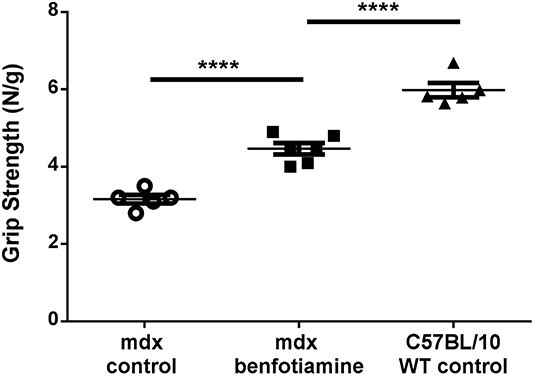
Benfotiamine increases grip strength in *mdx* mice. At 16 weeks of age, grip strength in benfotiamine treated *mdx* mice was improved towards wildtype (C57BL/10) levels. Grip strength was normalised to bodyweight. Graph shows mean ± SEM ^*^^*^^*^^*^P < 0.0001 *mdx* control n = 5 *mdx* benfotiamine n = 6 Wildtype (WT) C557BL/10 n = 5.

To determine if reduced dystrophic pathology observed with benfotiamine treatment translated to functional improvements in muscle strength and performance, forelimb grip strength was measured. In order to determine how the *mdx* control and *mdx* benfotiamine treated mice performed in comparison to healthy controls we included wildtype (C57/BL10) mice. Benfotiamine treated *mdx* had greater grip strength than control *mdx* (*P* < 0.0001) although the wildtype control mice were still significantly stronger (Fig. 4). These data show grip strength was improved towards wildtype levels in benfotiamine *mdx* mice.

**Figure 5:**
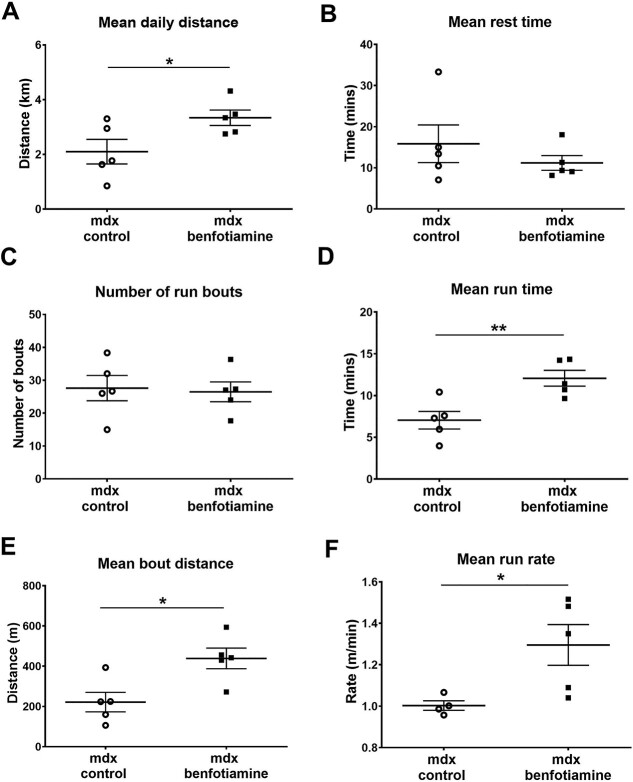
Benfotiamine improves voluntary exercise. Voluntary exercise was assessed in control *mdx* (n = 5) and benfotiamine *mdx* (n = 5) mice. Mouse were housed individually with access to an exercise wheel for 3 weeks (age: 16–19 weeks). A number of parameters were recorded; mean daily distance, mean run time, mean bout distance, mean run rate. Benfotiamine treated mice ran further each day (*P* < 0.05) (A), ran for a longer period of time (*P* < 0.01) (B), covered a greater distance each time they ran (*P* < 0.05) (C) and ran at a faster rate (*P* < 0.05) (D) than control *mdx*. The graphs show mean ± SEM. ^*^P < 0.05 ^*^^*^P < 0.01, *mdx* (n = 5) and benfotiamine *mdx* (n = 5).

To determine whether muscle performance was improved, mice were caged individually with access to an exercise wheel. The daily mean running distance was increased with benfotiamine treatment compared to control mice (*P* < 0.05) (Fig. 5A). There was no difference between the treatment groups in the mean rest time (Fig. 5B) or in the number of run bouts (Fig. 5C); but the benfotiamine treated *mdx* mice ran for longer (Fig. 5D) and covered more distance per exercise bout (*P* < 0.05) (Fig. 5E). The rate at which benfotiamine *mdx* mice ran was higher than controls (*P* < 0.05) (Fig. 5F). Overall, these data indicate that the improvements in dystrophic pathology observed with benfotiamine treatment are translated to improvements in muscle strength and performance.

### Benfotiamine reduces gene expression of pro-inflammatory markers in the quadricep muscle

**Figure 6:**
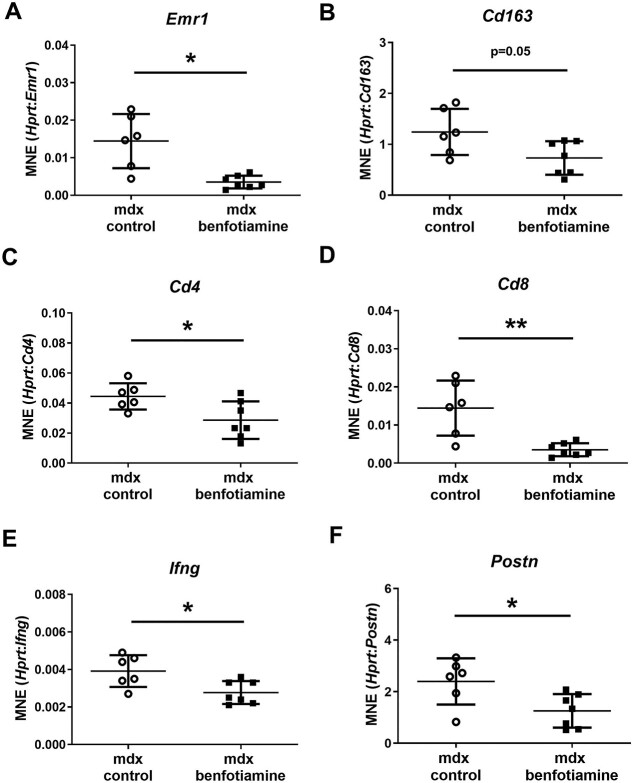
Benfotiamine down-regulates gene expression of pro-inflammatory markers in the quadriceps of *mdx* mice. Reduced gene expression by RT-qPCR was found in treated *mdx* for the pan-macrophage marker *Emr-1* (F480) (*P* < 0.05) (A), M2 macrophage marker (*Cd163*) (*P* = 0.05) (B), T lymphocyte markers *Cd4* (*P* < 0.05) (C), and *Cd8* (*P* < 0.01) (D). *Ifng* is a pro-inflammatory cytokine released from macrophages and naïve CD4 T cells (during Th2 differentiation), it’s expression was also downregulated with benfotiamine *mdx* (E) along with *Postn* (F) (*P* < 0.05)*,* an extracellular matrix protein and marker of inflammation and fibrosis. Graphs show mean ± SEM. ^*^Indicates *P* < 0.05 ^*^^*^P < 0.01. *mdx* (n = 6) and benfotiamine *mdx* (n = 7).

Since benfotiamine treated *mdx* had reduced areas of damage, which includes areas of fragmented sarcoplasm and an infiltration of inflammatory cell subsets, we investigated markers of inflammation. We found the pan-macrophage marker *Emr-1* (F480) (*P* < 0.05) (Fig. 6A), M2 macrophage marker (*CD163*) (*P* = 0.05) (Fig. 6B), T lymphocyte markers *Cd4* (*P* < 0.05) (Fig. 6C), and *Cd8* (*P* < 0.01) (Fig. 4D) were all down-regulated in benfotiamine treated muscle. Macrophages can present antigen to CD4+ cells causing Th2 differentiation and release of the pro-inflammatory cytokine IFNγ, activating additional pro-inflammatory macrophages. *Ifng* was downregulated with benfotiamine treatment (*P* < 0.05) (Fig. 6E). Periostin is a matricellular protein involved in the Th2 inflammatory response and fibrosis [36]. Periostin gene expression was also reduced in *mdx* treated with benfotiamine (*P* < 0.05) (Fig. 6F). Reduced expression of Th2 pro-inflammatory markers suggests benfotiamine impacts macrophages and T lymphocytes active in this pathway resulting in reduced inflammatory infiltrate.

### Benfotiamine regulates gene expression towards wildtype levels

**Figure 7:**
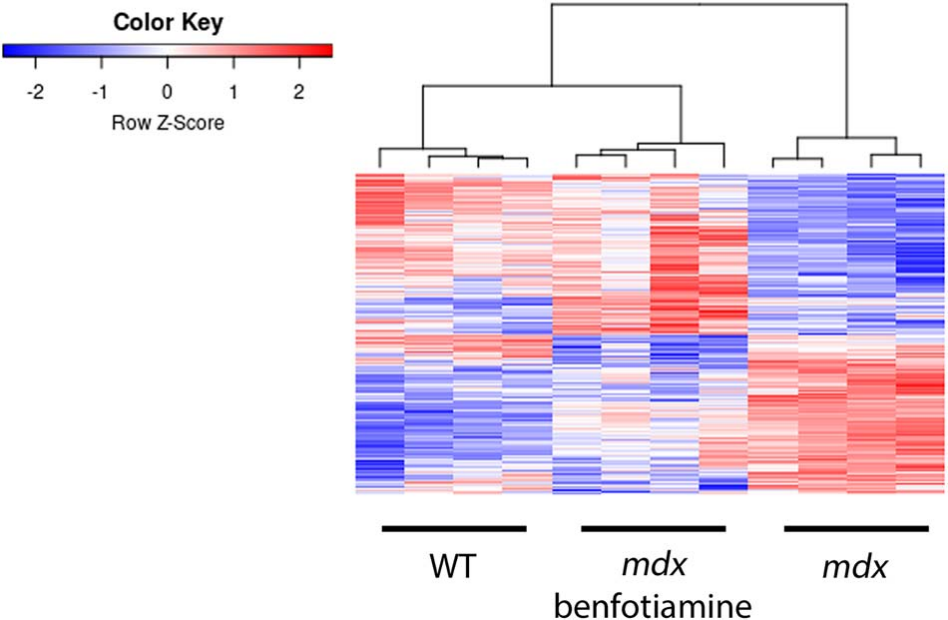
Heatmap of genes differentially expressed in *mdx* benfotiamine treated and *mdx* mice in the gastrocnemius muscle. Expression of differentially expressed (DE) genes (adj. *P*.Value < 0.05) was normalised across rows. The expression of DE genes in benfotiamine treated *mdx* shifts towards wildtype (WT) expression levels.

We investigated the changes in gene expression with benfotiamine treatment in the *gastrocnemius* muscle using RNAseq profiling. We found 5738 genes were differentially expressed in the *mdx* vs wildtype (WT) comparison (adjusted *P* value < 0.05) (Supplementary file 1). When *mdx* were treated with benfotiamine (*mdx* benfotiamine vs *mdx*) 494 genes were differentially expressed (adjusted *P* value < 0.05) (Supplementary file 2). A heatmap of these 494 genes shows their expression returning towards WT expression levels (Fig. 7). This is consistent with the improvement in grip strength towards WT levels and reduced muscle pathology measurements such as necrosis, central nuclei and myofibre size with benfotiamine treatment*.*

### Benfotiamine reduced expression of genes involved in the inflammatory response in dystrophic muscle

To investigate the genes sets impacted by benfotiamine treatment in *mdx*, we performed Gene Set Enrichment Analysis (GSEA) analysis. A summary of the Hallmark, GO and Curated gene sets identified using EGSEA can be found in Table 1. We found gene sets related to angiogenesis, myogenesis, hedgehodge signaling, oxidative phosphorylation, reactive oxygen species, inflammatory response and interferon pathways were impacted by benfotiamine treatment (Table 1). The top pathway found to be impacted by benfotiamine treatment in *mdx* was angiogenesis (adj. *P* < 0.001). Of the 52 genes listed in angiogenesis pathway, 12 were found to be have adj. *P* < 0.05 in benfotiamine treated *mdx*, 11 of which were downregulated (*Col1a1, Col1a2, Col4a5, Vav5, Itgav, Postn, Ccnd2, Col5a2, Lum, Col3a1, Col5a1*). These are predominantly genes found in the extracellular matrix and required for tissue remodelling. *Pdgfa* (platelet derived growth factor, alpha) is muscle specific growth factor required during muscle development and regeneration [37], it was the only gene in angiogenesis pathway upregulated (adj. *P* < 0.05) in benfotiamine *mdx*.

**Figure 8:**
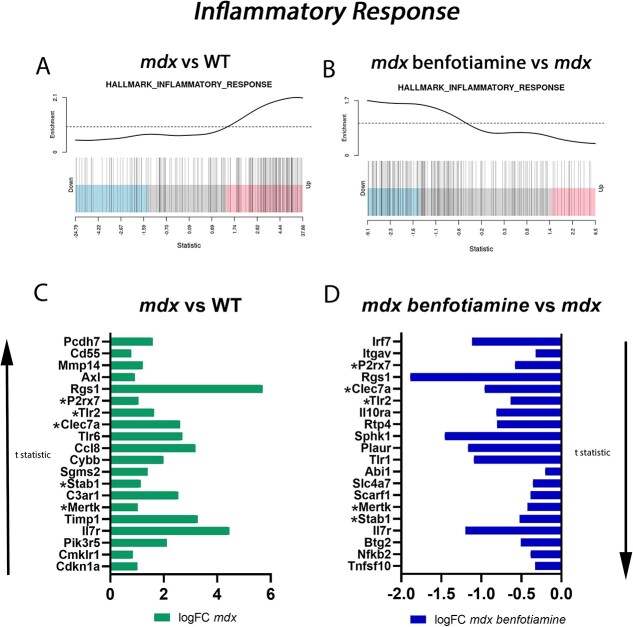
Gene set enrichment analysis (GSEA) shows benfotiamine reduces expression of *inflammatory response* genes in the gastrocnemius muscle of *mdx*. Barcode plots of the *inflammatory response* gene set in *mdx* vs WT show enrichment for upregulated genes (A). When *mdx* are treated with benfotiamine genes in the *inflammatory response* gene set are down regulated (B). (C) The top 20 genes ranked by t statistic, representing the most significantly upregulated inflammatory response genes *mdx* vs WT. (D) The top 20 genes ranked by t-statistic, representing the most significantly downregulated inflammatory response genes. ^*^Genes present in the top 20 in both comparisons.

Benfotiamine has been identified as an anti-inflammatory compound [19, 20, 38, 39]. From the EGSEA ranking, the *Inflammatory Response* (adj. *P* < 0.001) gene set (containing 200 genes) was down regulated in benfotiamine treated *mdx* muscle (Table 1). Barcode plots show *Inflammatory Response* genes tended to be upregulated in *mdx* vs WT mice (Fig. 8A) and downregulated in benfotiamine treated *mdx* vs untreated *mdx* (Fig. 8B) consistent with benfotiamine’s reported anti-inflammatory properties. We were interested to determine if the genes upregulated in *mdx* (vs WT) were downregulated with benfotiamine treatment. Analysis of the top 20 genes upregulated in *mdx* vs WT (Fig. 8C) and the top 20 genes downregulated in benfotiamine treated *mdx* vs *mdx* (Fig. 8D) (ranked by t statistic) revealed that *P2rx7, Tlr2, Clec7a, Stab1*, *Mertk* and *Il7r* were included in both gene lists suggesting benfotiamine treatment in *mdx* was reducing the inflammatory impacts of dystrophin loss. Other molecules known to play an important role in inflammation were also downregulated with benfotiamine treatment these included *IL10ra, Tlr1, Nfkb2*.

### Benfotiamine reduced expression of the myogenesis gene set in dystrophic muscle

**Figure 9:**
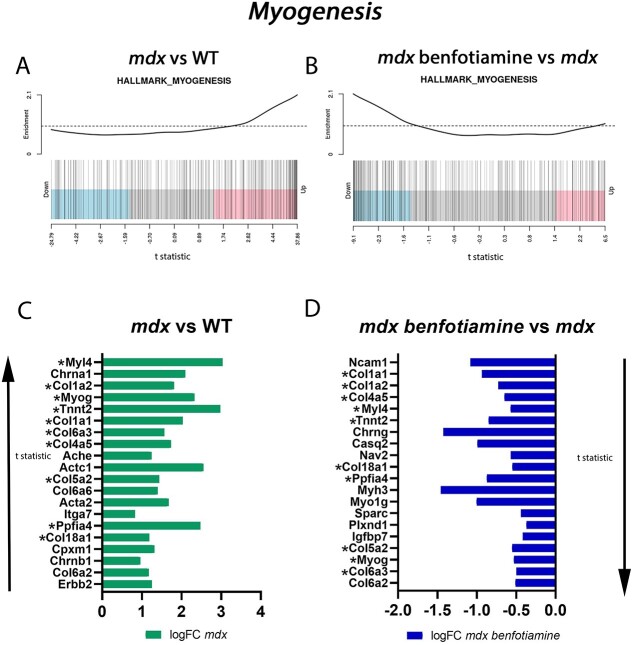
Gene set enrichment analysis shows benfotiamine impacts the *myogenesis* gene set in *mdx* gastrocnemius muscle. Barcode plots show the *myogenesis* gene set is upregulated in *mdx* vs WT (A) and down regulated in *mdx* treated with benfotiamine (B). The top 20 upregulated Hallmark myogenesis genes ranked by t statistic in *mdx* vs WT (C) and the top 20 downregulated myogenesis genes in benfotiamine treated *mdx* (D). ^*^Genes present in the top 20 in both comparisons.

The Hallmark *Myogenesis* gene set was downregulated in benfotiamine treated *mdx* (adjusted *P* value < 0.001). The myogenesis gene set is upregulated in *mdx* vs WT (Fig. 9A) and downregulated when *mdx* were treated with benfotiamine (Fig. 9B). Benfotiamine treated *mdx* muscle had fewer small myofibres relative to untreated *mdx* (Fig. 2D). Since smaller myofibres in *mdx* mice most often represent regenerating myofibres [40], this gene expression pattern is consistent with reduced contraction induced damage and regeneration in benfotiamine treated *mdx*. Sorting for t statistic, the top 20 genes upregulated in *mdx* vs WT (Fig. 9C) were compared with the top 20 genes downregulated in *mdx* benfotiamine (Fig. 9D). Eleven myogenic genes (*Myl4, Col1a2, Myog, Tnnt2, Col1a1, Col6a2, Col6a3, Col4a5, Col5a5, Ppfia4, and Col18a1)* were found to be differentially expressed in both comparisons (upregulated in *mdx* vs WT and downregulated in benfotiamine *mdx* vs *mdx*). Of these genes seven (*Col1a1, Col1a2, Col6a2, Col6a3, Col4a5, Col5a5, and Col18a1)* are collagen subunit genes expressed to make up proteins for assembly of collagen fibrils. Increased collagen expression in dystrophic muscle is an indication of fibrosis [41], upregulation of the myogenesis gene set could be reflective of increased fibrosis. In addition to the *Myogenesis* gene set being altered, we also found *Hedgehog* signalling pathway in benfotiamine treated *mdx* to be downregulated. This pathway is involved in skeletal muscle development in the embryo and during post-natal skeletal muscle regeneration following injury or in dystrophic muscle [42].

### Benfotiamine reduces gene expression of extracellular matrix fibrosis markers and utrophin

**Figure 10:**
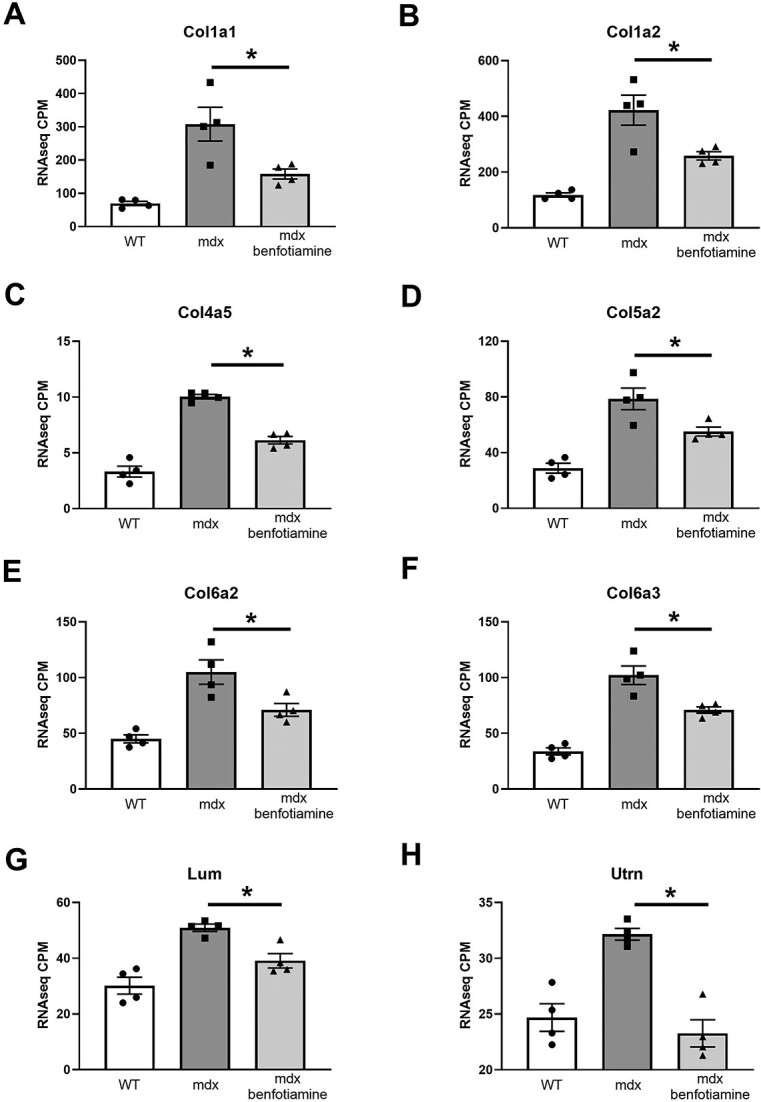
Benfotiamine treated *mdx* have reduced gene expression (RNAseq CPM) of extracellular matrix fibrosis markers and utrophin in the gastrocnemius muscle. Reduced gene expression of extracellular matrix proteins, in particular isoforms of collagen fibrils (*Col1a1* (A), *Col1a2* (B), *Col4a5* (C), *Col5a2* (D), *Col6a2* (E), *Col6a3*(F)) and the small leucine-rich (SLRP) proteoglycan *Lum* (G) were found in treated *mdx* showing expression more towards wildtype levels (*P* < 0.05). Utrophin is overexpressed in *mdx* mice compensating for loss of dystrophin to stabilise muscle membranes from contraction induced injury, benfotamine treated *mdx* have reduced expression of *Utrn* (*P* < 0.05). Graphs show mean ± SEM. ^*^Indicates *P* < 0.05 *mdx* (n = 4) and benfotiamine *mdx* (n = 4) CPM = counts per million.

**Figure 11:**
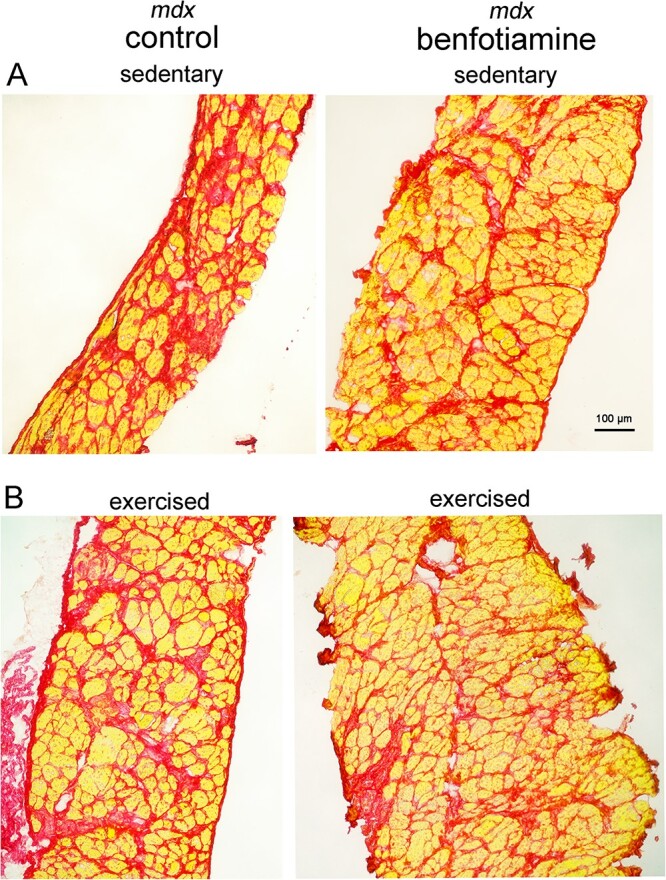
Benfotiamine treated *mdx* show reduced fibrosis in the diaphragm. Picrosirius red stains collagen (red) in diaphragm of sedentary and exercised *mdx* mice, myofibres appear yellow. Increased accumulation of collagen (red) is observed in *mdx* mice (sedentary/exercised) compared to benfotiamine treated *mdx*. No difference is apparent between sedentary and exercised mice. Scale bar 100 μm.

Considering genes in the *Inflammatory Response* and *Myogenesis* gene sets related to extracellular matrix and fibrosis were down regulated in benfotiamine treated *mdx* (Figs 8 and 9), we compared their gene expression relative to *mdx* control and WT mice with no dystropathology. Collagens [41] and the small leucine proteoglycan *Lum* [43] accumulate in dystrophic muscle. Extracellular matrix genes down regulated in benfotiamine *mdx* and expressed more closely to WT levels included: *Col1a1, Col1a2, Col4a5, Col5a2, Col6a2* and *Col6a3* (Fig. 10). In benfotiamine *mdx*, these fibrotic gene markers were expressed 1.5–2-fold higher than in WT mice, while in untreated *mdx* their expression was 2–4-fold higher than in WT mice. To further support that benfotiamine reduces markers of fibrosis, we performed collagen staining (Picro Sirius Red) in the diaphragm of benfotiamine treated *mdx* mice (sedentary and exercised) (Fig. 11). Accumulation of collagen (red) is evident in diaphragm in replacement of myofibres in *mdx* mice (sedentary and exercised), this is reduced in benfotiamine treated *mdx* mice in both sedentary and exercised mice.

## Discussion

Benfotiamine, a lipid soluble analogue of vitamin B1, is used as an anti-inflammatory to treat patients with diabetic neuropathy. Cycles of muscle degeneration and regeneration are pathological hallmarks of Duchenne muscular dystrophy (DMD), with inflammation and fibrosis as major contributing factors. One of the few treatments for DMD patients are corticosteroids (prednisone/prednisolone/deflazacort) acting to exert immunosuppressive and anti-inflammatory effects, reduce fibrosis, muscle cell proteolysis, upregulate utrophin, and improve muscle strength and functional outcome [44–47]. In this study, we found that benfotiamine had a protective role in the *mdx* mouse, reducing dystropathology by reducing expression of pro-inflammatory markers and gene sets related to inflammation, muscle regeneration and fibrosis. Improvements in voluntary running capacity is considered a strong predictor of disease severity, and benfotiamine treated *mdx* displayed improved ability to exercise and improved grip strength. The histopathological changes associated with disease progression in DMD include many fibres with central nucleation, variation in fibre diameter, extensive necrosis, chronic inflammation, fat deposition and tissue fibrosis [48–50]. We found *mdx* treated with benfotiamine showed improvements in disease progression by having reduced myofibres with central nucleation and necrosis.

**Table 1:** Summary of gene set enrichment analysis with Hallmark, GO and curated gene sets (MSigDB) on benfotiamine treated *mdx* vs *mdx* using EGSEA.

Hallmark pathway	Rank	Adjusted *P* value	Expression
Angiogenesis	1	*P* < 0.001	DOWN
Myogenesis	2	*P* < 0.001	DOWN
Apical Junction	3	*P* < 0.001	DOWN
Epithelial mesenchymal transition	4	*P* < 0.001	DOWN
Mitotic spindle	5	*P* < 0.05	DOWN
G2M checkpoint	6	*P* < 0.05	DOWN
Hedgehog signaling	7	*P* < 0.01	DOWN
Myc targets V1	8	*P* < 0.001	UP
Complement	9	*P* < 0.001	DOWN
Androgen response	10	0.2357	DOWN
Estrogen response late	11	*P* < 0.001	DOWN
Kras signalling	12	*P* < 0.001	DOWN
DNA repair	13	*P* < 0.01	UP
Apical surface	14	0.2240	DOWN
Oxidative phosphorylation	15	*P* < 0.001	UP
Reactive Oxygen species pathway	16	0.1904	UP
UV response	17	0.0526	DOWN
Kras signalling	18	*P* < 0.001	DOWN
E2F Targets	19	0.1849	DOWN
Allograft rejection	20	*P* < 0.001	DOWN
Inflammatory response	21	*P* < 0.001	DOWN
Interferon alpha response	22	1.64E-05	DOWN

We used global gene expression profiling to determine the gene sets changed in benfotiamine *mdx* that could contribute to reduced disease severity. We found pathways related to inflammation (*Inflammatory Response*) and myogenesis (*Myogenesi*s and *Hedgehog Signalling*) were downregulated in benfotiamine *mdx*. Corticosteroid treatment reduces inflammation, and degeneration of myofibres [51]. In benfotiamine treated *mdx,* there was reduced activation of the *Inflammatory Response* pathway in the gastrocnemius*,* and in the quadriceps we found reduced dystropathology and reduced expression of the pro-inflammatory gene markers *Emr1* (F4/80)*, Cd4, Cd8, Ifng, Pstn* and the pro-regenerative macrophage marker *Cd163. Emr1* (F4/80)*, Cd4, Cd8,* and *Ifng* are inflammatory markers for macrophages (*Emr1),* CD4+ T cells (*Cd4),* CD8+ T cells (*Cd8).* Periostin (*Pstn*) is a matricellular protein involved in the Th2 inflammatory response, its release from fibroblasts exacerbates Th2 inflammation and fibrosis related chronic inflammation [36]. Reduced expression of these markers demonstrates benfotiamine treatment reduces *mdx* pathology by targeting inflammatory genes. Based on this information we have attributed the reduced pathology in the *quadriceps* muscle of treated *mdx* to downregulation of genes related to inflammation, myogenesis and muscle fibrosis as evident in alterations of gene sets found in the gastrocnemius muscle.

Inflammation is a secondary process in dystrophic muscle resulting from dystrophin deficiency. Leukocytes infiltrate to repair contraction-induced damaged myofibres, these include neutrophils, eosinophils, macrophages, natural killer, regulatory T cells, and CD4+/CD8+ T cells [52, 53]. The primary role of the pro-inflammatory infiltrate is to clear damaged myofibres causing necrosis. The major components in the inflammatory infiltrate in necrotic myofibres include F4/80 macrophages and CD4/CD8 T cells [52, 53]. We found the *Inflammatory Response* genes were downregulated in benfotiamine *mdx* muscle*.* This pathway was activated in *mdx* vs WT. In this pathway, we found genes such as *P2x7r,* and *Tlr2* receptor that are part of the inflammasome [54, 55] are downregulated in treated *mdx* muscle. These molecules are involved in activating caspase 1 to drive cleavage of the pro-inflammatory cytokines pro-IL-1β and pro-IL-18, and gasdermin [54, 55]. Cleavage of gasdermin induces pyroptosis and allows release of IL-1β and IL-18 from the cytosol to further induce inflammation [56]. Accumulation of adenosine triphosphate (ATP) occurs at sites of tissue injury and inflammation [57]. *P2rx7* is expressed by cells of the adaptive and innate immunity systems and is activated by ATP mediating the activation of the NLRP3 inflammasome [57]. We have shown previously that exacerbation of *mdx* pathology by exercise upregulated many genes that are part of the inflammasome including *Tlr2, Tlr6*, and *P2rX7* [43]. The effect of benfotiamine on these molecules suggests its effects are impacting pathways that are part of the inflammasome.

A possible mechanism for benfotiamine to impact inflammation and pathways associated with, could be through dampening of advanced glycation end products (AGEs). Benfotiamine can reduce oxidative damage and inflammation by accelerating precursors in the pentose phosphate pathway, reducing advanced glycation end products (AGEs) [18]. AGE’s can bind to the receptor for advanced glycation end products (RAGE), on mononuclear infiltrates in dystrophic muscle, to induce inflammation [19]. Targeting RAGE’s in *mdx* mice has anti-inflammatory effects and improves muscle regeneration [19], similarly benfotiamine could be impacting on inflammation in *mdx* by reducing AGEs and their receptor binding (to RAGEs).

The *Myogenesis* gene set was downregulated in benfotiamine treated *mdx*. In this gene set *Myog*, one of the myogenic regulatory factors was reduced. Evidence that markers of inflammation and myogenesis are reduced in treated *mdx* suggests benfotiamine dampens damage in dystrophic skeletal muscle with a reduced need for myogenic repair. Other terminal differentiation markers of myogenesis downregulated in treated *mdx* include *Ncam1* and *Myl4. Ncam1* and *Myl4,* all are markers of embryonic skeletal muscle development, their activation in post-natal muscle is driven by skeletal muscle injury and regeneration [58, 59].

Benfotiamine treatment *mdx* had an increased capacity for exercise by running further and faster than untreated mice. We believe this is attributed to less damage and reduced markers of inflammation and fibrosis in benfotiamine treated *mdx.* The progressive accumulation of collagen and related ECM (extracellular matrix) proteins and the apparent dysregulation of matricellular proteins play a debilitating role in DMD, with the progressive loss of muscle fibres and their replacement with non-contractile fibrotic tissue correlating with reduced motor function [60]. Excessive accumulation of ECM components is an indicator of the decline in muscle strength [61]. The observation of reduced accumulation of collagen staining in diaphragm of benfotiamine treated *mdx* warrant the need for further investigation into the signalling pathways benfotiamine targets in this muscle to determine if benfotiamine is impacting inflammation which leads to reduced fibrosis or if this treatment is directly acting on extracellular matrix pathways. Respiratory failure and cardiac failure are the leading cause of death in DMD because of the dystrophic muscle damage. Treatments impacting the diaphragm to reduce fibrosis are optimal candidates for clinical setting for DMD and other neuromuscular conditions where fibrosis is evident in diaphragm to impact quality of life and survival.

Utrophin is upregulated during active muscle regeneration and is upregulated in *mdx* muscle to compensate for the loss of dystrophin providing a milder pathology than DMD [62]. However, expression is higher in more severe dystrophinopathy patients [63]. Consistent with reduced central nucleation observed in the benfotiamine-treated muscle, we did not observe an increase in utrophin in *mdx* benfotiamine, suggesting benfotiamine treated *mdx* have less severe pathology.

This is the first study to assess the potential of benfotiamine as a treatment for neuromuscular conditions such as DMD. The pre-clinical data generated from this study is promising and warrants further investigation. The limitations of this study are the low sample size for each mouse cohort. The exercise cages require mice to be individually caged for accurate exercise measurements reducing the number of mice able to run simultaneously. In addition, mice that did not run more than 1 km per night were excluded from the study reducing numbers further. Future experiments could focus on exercise cohort to build animal numbers and investigate metabolic pathways, specifically transketolase in the pentose phosphate pathway to determine if benfotiamine is impacting this pathway to reduce AGE’s and infiltration of inflammatory immune cells. A more detailed investigation of the immune cells could also be tested using flow cytometry to identify alterations in specific immune cell subtypes (T cells, B cells, Innate cells) in muscle, spleen, thymus and blood of mice treated with benfotiamine.

Overall, our data suggests that benfotiamine reduces DMD pathology and these effects are mediated by reducing inflammation and fibrosis. Due to its excellent safety profile and use in clinical trials for other diseases [15, 27–29, 31], benfotiamine could be transitioned rapidly into a clinical setting as a combinatorial therapy with current steroid treatment or in addition to emerging therapies, thus providing benefits to many DMD patients.

## Materials and methods

### Animals

Animal experiments were approved by the University of Melbourne Animal Ethics Committee (AEC) and the Murdoch Children’s Research Institute AEC. Mice were purchased from The *Animal Resources Centre (Perth, Western Australia) and* cared for according to the “Australian Code of Practice for the Care of Animals for Scientific Purposes” published by the National Health and Medical Research Council (NHMRC) Australia. They were housed under a 12-hour light/dark cycle with food and water provided *ad libitum*.

### Mice cohorts

Benfotiamine was administered to *mdx* mice through their food. Benfotiamine (Sigma Aldrich) was added to standard mouse chow (Specialty Feeds, Glen Forrest, Western Australia) at a concentration of (10 mg/kg/day). Benfotiamine diet was fed to three male *mdx* mice cohorts for 15 weeks (from 4 weeks of age) and compared to control *mdx* mice who were fed standard chow. The three cohorts consisted of Cohort 1) Sedentary group (*mdx* control n = 6, *mdx* benfotiamine n = 7), Cohort 2) Exercise group (*mdx* control n = 5, *mdx* benfotiamine n = 5) and Cohort 3) Grip strength group (*mdx* control n = 5, *mdx* benfotiamine n = 6, wildtype C57BL/10 n = 5). The exercise group were housed in individual cages with access to exercise wheel from 15 weeks of age for 3 weeks. A timeline for mouse cohorts can be found Fig. 1A. Exercise activity was recorded as rotation of the wheel every 1 min as described previously [64] after a week of acclimatisation. Mice that ran less than 1 km/day per day were excluded from the study, these mice were equally distributed for treatment. RNAseq was performed on the exercised mice (Cohort 2). Mice genotypes, treatments and exercise intervention were blinded to those performing the following measurements.

### Forelimb grip strength measurements

Forelimb grip strength was measured weekly from 4 weeks of age using a BIO-GS3 grip strength meter (Bioseb In Vivo Research Instruments). The procedure was performed as per the Treat-NMD standard operating procedure (http://www.treat-nmd.eu/downloads/file/sops/sma/SMA_M.2.1.002.pdf). The force of the pull (N) prior to release was recorded and the mouse was placed back in its cage and allowed to recover for 5–9 min before repeating the test another 4 times. Data presented was an average of the 5 attempts.

### Tissue harvest

Tissue was harvested from the *Cohort 1—sedentary mice* for dystrophic pathology measurements (Fig. 1A) and from *Cohort 2—exercise mice* for RNAseq transcriptome gene profiling (Fig. 1A). Sedentary *mdx* mice were anaesthetised with isofluorane, blood was obtained via cardiac puncture. All mice were humanely euthanized by cervical dislocation. Quadriceps (*sedentary cohort*) and gastrocnemius (*exercise cohort*) muscles were snap frozen in liquid nitrogen for RNA isolation and the contralateral Quadriceps and diaphragms were mounted in 5% tragacanth (w/v) and frozen in liquid nitrogen cooled isopentane for histology. Muscles were stored at −80°C until required.

### Histology and immunostaining

Frozen quadricep sections were thawed and blocked in 10% (v/v) donkey serum (Millipore, Billerica, Massachusetts, USA) diluted in wash buffer (0.1% Tween, 0.5% BSA in 1xPBS) and stained with anti-laminin α2 (Santa Cruz Biotechnology) diluted in wash buffer overnight at 4°C. Sections were washed in wash buffer and stained with anti-rat AlexaFluor594 and donkey anti-mouse IgG secondary antibodies (diluted in wash buffer) in the dark for 90 min. Sections were washed and stained with 1 μg/μl Hoechst (Life Technologies, Carlsbad, California, USA) Sections. were imaged on a Zeiss Axio Imager M1 upright fluorescent microscope with an AxioCam MRm camera running AxioVision software V4.8.2.0 (Carl Zeiss, Oberkochen, Germany). Frozen diaphragm sections were stained for PicroSirius Red at University of Melbourne Histology Platform (Melbourne, Australia) and imaged as above using Brightfield camera.

### Measures of histopathology

In all histological and morphometric assessments, the total cross-section of the quadriceps was analyzed, therefore a minimum of 2500 myofibres per animal were assessed. The treatment groups for these experiments were blinded to prevent any experimenter bias.

### Minimum Feret’s diameter

Sections stained with laminin α2 were used to determine myofibre diameter. Images were analysed in Image J version 1.48G (U. S. National Institutes of Health, Bethesda, Maryland, USA). The image threshold was set and minimum Feret’s diameter was calculated according to the Treat- NMD standard operating procedure “Quantitative determination of muscle fibre diameter” (http://www.treat-nmd.eu/downloads/file/sops/dmd/MDX/DMD_M.1.2.001.pdf).

### Central nucleation

The count tool in Image J was used to count the number of myofibres with centrally located nuclei as a proportion of the total number of myofibres for the entire cross-sectional area of each quadricep. Each was expressed as a percentage and averaged over each treatment group.

### Measurement of anti-IgG positive damaged myofibres

To determine the percentage of damaged myofibres in the quadriceps, the muscle was transversely sectioned and stained with anti-laminin α2 to stain the basement membrane of the myofibres and anti-IgG (Alexa Fluor 488), which stains damaged myofibres with weakened sarcolemma [65]. Anti-IgG positive myofibres for the entire quadricep cross-sectional areas were counted manually and expressed as a percentage of the total myofibre number.

### Measurement of skeletal muscle necrosis

To assess the amount of necrosis present in the quadriceps muscle the transverse sections were stained with haematoxylin and eosin (H&E). Using Image J the total area of the muscle cross-section was calculated. Areas of necrosis were defined based on the presence of infiltrating inflammatory cells and areas of degenerating myofibres with fragmented sarcoplasm according to Treat-NMD standard operating procedure (http://www.treat-nmd.eu/downloads/file/sops/dmd/MDX/DMD_M.1.2.007.pdf). The amount of necrosis was expressed as a percentage of the total quadriceps area [64].

### Creatine kinase assay

For measure of serum creatine kinase (CK) activity, bloods were collected via cardiac puncture and centrifuged at 12 000 × g for 10 min at 4°C to obtain serum. Serum CK activity was determined on the first thaw using the commercially available reagent, N-Acetyl Cysteine (Thermo Scientific, Waltham, Massachusetts, USA) as per manufactures instructions. The change in absorbance was recorded kinetically at 340 nm for three minutes (measured in 20 s intervals) at 37°C using a Paradigm Detection Platform (Beckman Coulter, Brea, California, USA).

### RNA extraction, cDNA synthesis and qRT-PCR

RNA was extracted with TriReagent (Sigma Aldrich) followed by purification and DNase treatment using the SV Total RNA Isolation System (Promega). cDNA was synthesized from 1 μg total cellular RNA with MML-V Reverse Transcriptase (Promega). Gene expression was quantitated using qPCR as previously described [66]. Oligonucleotide sequences are presented in Table 2. Data are expressed as the mean of normalized expression (MNE) to the housekeeper hypoxanthine-guanine phosphoribosyltransferase (*Hprt*).

**Table 2:** Primer sequences used for qRT-PCR.

Primer Name	Sequence Forward (5′-3′)	Sequence Reverse (5′-3′)
*Cd4*	CGTGCTGGGTGGCTCCTTCG	CTTCTGCATCCGGTGGGGGC
*Cd8* *Cd163*	GGCTCAGTGAAGGGGACCGGA TGCGCCGACGTGTTCCGAAG	AGCGGCCTGGGAACATTTGCAAAA GCTGGCCACTTGCTATGCAGGG
*Emr1*	ACAGCCACGGGGCTATGGGA	GCACCCAGGAGCAGCCCCAG
*Hprt*	GATTAGCGATGATGAACCAGGTT	TCCAAATCCTCGGCATAATGAT
*Ifng*	AGTTTGAGGTCAACAACCCACAGGT	CCACCCGAATCAGCAGCGA
*Postn*	AACCAAGGACCTGAAACACG	CAACACCATTTGTGGCAATC

### Bulk population RNAseq

For RNAseq, gastrocnemius muscles (n = 4 per group) were snap frozen in liquid nitrogen, pulverized using a liquid nitrogen-cooled tissue grinder and RNA extracted with TRIzol, followed by purification using Direct-zol RNA Microprep kit spin columns (Zymo Research) according to the manufacturer’s instructions. RNA samples were quality controlled and sequenced at the Translational Genomics Unit, Murdoch Children’s Research Institute. Libraries were constructed using Illumina Stranded mRNA Prep kits and sequenced using a NextSeq 500 to obtain ~20 × 10^6^ 75 bp paired-end reads per sample. Reads were aligned to the mouse reference genome (GRCm38) using an RNAseq pipeline that incorporated FastQC quality control, adaptor trimming with Trimmomatic [67] mapping with STAR [68], summarizing reads over genes with featureCounts [69], and MultiQC [70] to summarize the analyses. Downstream analyses and identification of differentially expressed genes used the EdgeR Bioconductor package [71]. Genes with expression levels of at least one count per million in at least four samples were kept for further analysis. The data were TMM normalized and voom transformed. Differential expression was identified with robust paired moderated t tests using limma [71]. Gene set enrichment analysis used the EGSEA and EGSEAdata packages [72]. Graphical visualisations used the gplots, tidyverse and ggplot2 packages. All gene set analyses were completed in February 2020.

### Statistical analyses

Statistical analyses used GraphPad Prism Version 7.04. A Student’s t-test (Unpaired two-tailed) was used to compare differences between groups*.* The mean and standard error of the mean (±SEM) is presented, a *P* value of < 0.05 was considered statistically significant.

##  

*Conflict of interest statement*: The authors have declared no conflict of interest.

## Funding

This work was supported by Muscular Dystrophy Australia, Murdoch Children’s Research Institute and the Victorian Government’s Operational Infrastructure Support Program. This work was supported by grants from the National Institutes of Health (R01 AR048179 to R.H.C., T32 AR059033 and F32 AR069469 to E.M.G.) and the Muscular Dystrophy Association USA (274143 and 416364 to R.H.C.). S.R.L. was supported by a National Health and Medical Research Council of Australia research fellowship (GNT1043837).

## Supplementary Material

Supplementary_File_1_mdx_control_vs_WT_control_ddae066

Supplementary_File_2_mdx_benfotiamine_vs_control_ddae066
